# Introduction of HPV testing for cervical cancer screening in Central America: The Scale-Up project^☆^

**DOI:** 10.1016/j.ypmed.2020.106076

**Published:** 2020-06

**Authors:** Francesca Holme, Jose Jeronimo, Francisco Maldonado, Claudia Camel, Manuel Sandoval, Benito Martinez-Granera, Mirna Montenegro, Jacqueline Figueroa, Rose Slavkovsky, Kerry A. Thomson, Silvia de Sanjose, the Scale-Up project team

**Affiliations:** a PATH, Seattle, WA, USA; b José Jeronimo Consulting, Damascus, MD, USA; c Movicáncer, Managua, Nicaragua; d Ministry of Public Health and Social Assistance, Guatemala City, Guatemala; e Asociación Hondureña de Planificación de Familia, Tegucigalpa, Honduras; f Instancia por la Salud y el Desarrollo de las Mujeres, Guatemala City, Guatemala; g Secretary of Health, Tegucigalpa, Honduras

**Keywords:** Screening, HPV, Cervical cancer, Thermal ablation, Health information system

## Abstract

The Scale-Up project introduced vaginal self-sampling and low-cost human papillomavirus (HPV) testing as the primary approach for cervical cancer screening in selected public health centers in Guatemala, Honduras, and Nicaragua. We evaluate the country-specific accomplishments in screening: target-coverage, triage, and treatment. Between 2015 and 2018, cervical cancer screening was offered to women at least 30 years of age. Triage of HPV-positive women was based on visual inspection with acetic acid or Pap. Aggregated data included total women screened, use of self-sampling, age, time elapsed since last screening, HPV results, triage tests, triage results, and treatment. A total of 231,741 women were screened for HPV, representing 85.8% of the target populations within the project. HPV positivity was lower in Guatemala (12.4%) compared to Honduras and Nicaragua (14.5% and 14.2%, respectively, *p* < 0.05). A follow-up triage test was completed for 84.2%, 85.8%, and 50.1% of HPV-positive women in Guatemala, Nicaragua, and Honduras, respectively. Of those with a positive triage test, 84.7%, 67.1%, and 58.8% were treated in Guatemala, Nicaragua, and Honduras, respectively. First-time screening was highest in Nicaragua (55.8%) where self-sampling was also widely used (97.1%). The Scale-Up project demonstrated that large-scale cervical cancer screening and treatment intervention in a high-burden, low-resource setting can be achieved. Self-sampling and ablative treatment were key to the project's achievements. Data monitoring, loss to follow-up, and triage methods of screen- positive women remain critical to full success.

## Highlights

• Central America successfully implemented HPV testing in public sector clinics.
• HPV testing using self-sampling holds great promise to increase coverage of cervical cancer screening.
• Follow-up of screen positives is a critical component step for an effective screening program.
• Thermal ablation of precancerous lesions may be critical in increasing treatment coverage.
• Basic health information systems are required for monitoring and mitigating loss to follow-up.

## 1 Introduction

In 2018, an estimated 570,000 women worldwide developed cervical cancer (Bray et al., 2018), a disease that can be prevented by human papillomavirus (HPV) vaccination or by early detection and treatment of precancerous lesions. Approximately 300,000 women die annually from cervical cancer, with 90% of this burden occurring in the world's poorest countries (Bray et al., 2018; Murillo et al., 2016). Cervical cancer diagnosis peaks when women are in their 40s, a time when they are often the anchor of their communities and provide important economic and social stability to their families (Schiffman et al., 2016). Compelling progress in primary prevention is now being made through HPV vaccination and screening, prompting the World Health Organization (WHO) to declare that elimination of cervical cancer as a public health problem is feasible. While this call to action has been lauded and answered by numerous organizations (Union for International Cancer Control (UICC) website, n.d.; American Cancer Society Cancer Action Network website, n.d.),limited effectiveness of screening programs remains a major challenge in low- and middle-income countries (Murillo et al., 2008) (LMICs).Self-sampling and screening with HPV testing bring tremendous potential to increase efficacious coverage to detect cervical intraepithelial neoplasia (CIN) lesions (Arbyn et al., 2018). Arrossi et al. have recently shown using HPV testing that self-sampling was a well-accepted approach to detect high-grade pre-neoplastic lesions, however, a high proportion of loss to follow-up between screening and treatment was observed, reducing the overall prevention impact of screening. (Arrossi et al., 2019)

Cervical cancer continues to be a significant health problem in Guatemala, Honduras, Nicaragua, and El Salvador with age-adjusted incidence rates ranging from 18.5 to 21.2 per 100,000, considerably higher than rates for the United States and Canada (Bray et al., 2018; International Agency for Research on Cancer website, 2018). These four countries have historically invested in cervical cancer screening but have mostly applied opportunistic approaches using Pap smear, resulting in low coverage and an unmeasured and limited impact on incidence and mortality (Murillo et al., 2008).

In 2011, PATH's Screening Technologies to Advance Rapid Testing for Cervical Cancer Prevention—Utility and Program Planning (START-UP) project showed that screening with cervical or vaginal HPV testing performed better than visual inspection with acetic acid (VIA) or Pap testing in resource-limited settings (Jeronimo et al., 2014). START-UP introduced *care*HPV, a low-cost test specifically designed for use in low-resource settings using either cervical or vaginal samples (Jeronimo et al., 2014).

Building upon this evidence, PATH worked with Central American ministries of health (MOHs) to introduce a new model of cervical cancer screening under the Scale-Up project. Scale-Up was implemented in selected areas in Guatemala, Honduras, and Nicaragua from 2015 to 2018. Essential elements of the project's implementation were: a) raising awareness at the community level, b) facilitating adoption and encouraging procurement of HPV testing, c) using vaginal self-sampling for test specimens to facilitate rapid screening uptake and d) developing indicators to be monitored throughout the activity. El Salvador followed a different timeline for screening and implemented a different treatment algorithm; thus, results from this country are reported elsewhere (Maza et al., 2017).

Here we report on the country-specific accomplishments in screening target coverage, triage, and treatment indicators in Guatemala, Honduras, and Nicaragua. Collectively, these results can inform decision-making in other low-resource settings, as countries consider more efficient and effective models for secondary prevention of cervical cancer.

## 2 Methods

### 2.1 Partner roles, project implementation, and participant eligibility

**Fig. 1:**
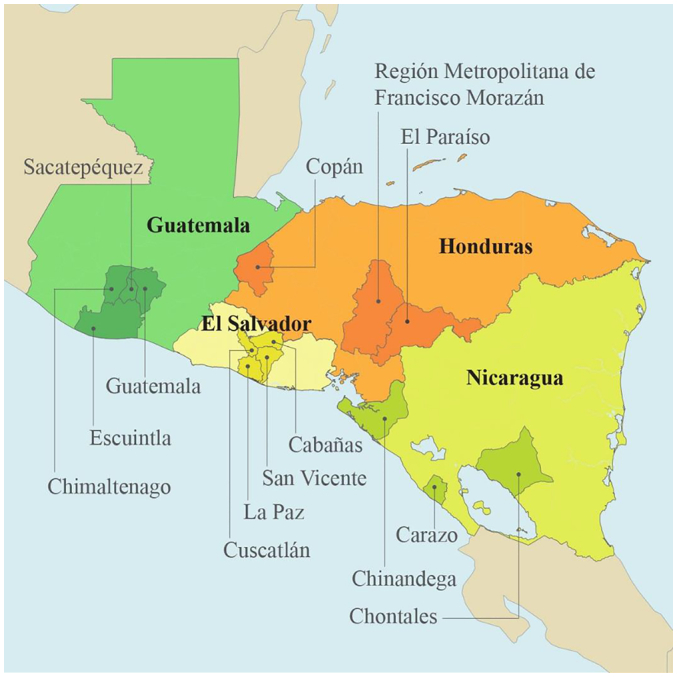
Map of countries and target areas in the Scale-Up Project.

The MOHs of Guatemala, Honduras, and Nicaragua defined geographic target areas (Fig. 1) and screening algorithms with technical assistance from PATH. PATH partnered with local non-governmental organizations to aid the MOHs in implementing the project through planning, advocacy, coordination of screening and follow-up, development of community outreach strategies, and data collection. PATH coordinated the project across countries, consolidated data, and performed data analyses. HPV tests were provided to the countries through project funds.

**Fig. 2:**
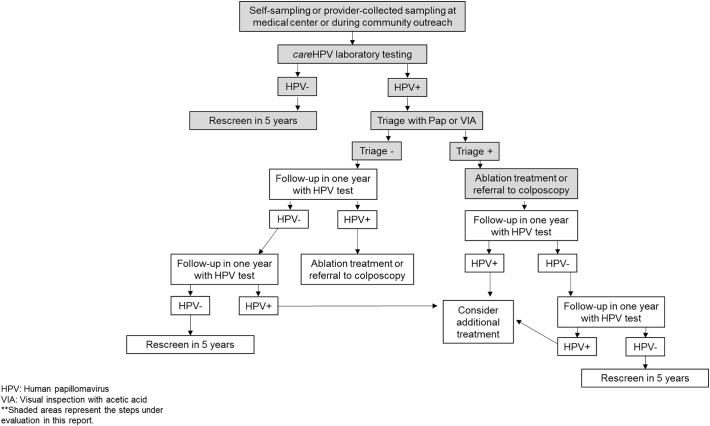
General algorithm for the screening intervention using HPV as primary screening test under the Scale-project in Guatemala, Honduras and Nicaragua.

The Scale-Up project was planned in three phases: preparation, pilot testing, and expansion; project details have been described previously (Jeronimo et al., 2016). Each country adapted its own guidelines for cervical cancer screening based on WHO 2013 recommendations (World Health Organization (WHO) website, n.d.-a) to include HPV testing, specifying target age groups, management of HPV screen-positive women, and follow-up (Table 1 and Fig. 2). Eligibility for screening began at age 30 in all three countries, and included women up until age 59, 64, and 65 years in Nicaragua, Honduras, and Guatemala, respectively. Women were invited to participate in screening activities through different contact points, mainly primary health care centers and community outreach.

**Table 1:** Characteristics of the Scale-Up Project by country.

Country	PATH's local partner organization	Project departments	Total estimated population in departments^a^	Number of HPV tests delivered	Management algorithm for HPV positive women	Self-sampling available
Guatemala	La Instancia por la Salud y el Desarrollo de las Mujeres (ISDM)	Guatemala central Guatemala Sur Chimaltenango Sacatepéquez	496,407	93,000	Visual inspection with acetic acid (VIA), or cervical cytology (pap) as secondary option	Yes
Honduras	Asociación Hondureña de Planificación de la Familia (ASHONPLAFA)	Copán El Paraíso Metropolitana Francisco Morazan	448,948	83,000	VIA	Yes^b^
Nicaragua	Movicáncer	Carazo Chontales Chinandega	141,637	83,000	Pap, or VIA as secondary option	Yes
El Salvador^c^	Basic health international (BHI)	San Vicente Cuscatlán Cabañas La Paz	132,173	20,000	VIA to determine eligibility for ablation, and treat all HPV positive	No

a Based on the most recent census data.

b Offered starting in 2017 in the largest department only, the metropolitan region of Tegucigalpa.

c The El Salvador Ministry of Health followed a different protocol on community outreach and clinical management compared to other scale-up countries and thus results are presented elsewhere. Tests for El Salvador were donated by QIAGEN.

### 2.2 Screening and treatment methods

The screening assay used was *care*HPV (QIAGEN, Hilden, Germany), a signal-amplification batch diagnostic test for high-risk HPV DNA detection. The test allows qualitative detection of 14 high-risk types of HPV DNA in cervical and vaginal specimens. QIAGEN and PATH trained local laboratory microbiologists, cytologists, and technicians to run the test. PATH also developed quality assurance guidelines (Beylerian et al., 2018; PATH, 2018).

Guatemala and Nicaragua selected self-sampling using *care*HPV as the primary screening strategy. Health workers offered HPV screening tests free of charge in a mixture of outreach models, either at health clinics (the majority) or community-based locations (e.g., homes, markets, worksites); less commonly, providers collected specimens during a pelvic exam at clinic sites. In Honduras, self-sampling was promoted in one province only, the metropolitan region of Tegucigalpa, from 2017 onward. In all three countries, HPV samples were sent to referral laboratories within approximately one week of collection, and women could collect the test result in one month from the clinic. Women who provided a sample outside of the clinic also had an option to receive their HPV test results from a community health worker. A triage appointment at the primary health care center was scheduled for HPV-positive women. Triage results and treatment eligibility for the HPV-positive women were evaluated, using primarily VIA in Guatemala and Honduras, and cervical cytology in Nicaragua. For women outside of the country's target age range for HPV testing, or if HPV tests were temporarily not available at the primary health care center, screening by VIA or Pap was offered.

Women with triage-positive lesions eligible for ablative treatment were treated with cryotherapy, although thermal ablation was introduced and subsequently used in Guatemala in 2017 and in Honduras in 2018. If not eligible for ablation because the transformation zone was not fully visible or the lesion occupied >75% of the transformation zone, women were referred to a higher level of care.

### 2.3 Definition of indicators

Indicators were collected and compiled in an aggregated and anonymous form that was sent at least quarterly to PATH. In addition to the number of tests provided per country, the information collected included (1) women screened with HPV testing, (2) women providing self-collected samples, (3) women in the target age range, (4) time elapsed since last cervical cancer screening, (5) HPV-positive women, (6) women receiving a triage test, (7) women positive in triage, and (8) women treated for precancer.

Each country had a pre-established number of tests to be used in the project. Target-coverage was estimated as the number of women screened divided by the number of tests allocated to a country during the project The estimates cannot correct for repeated tests during the period.

### 2.4 Data analysis

Data were analyzed using Excel and Stata/SE 15.0 Whenever appropriate, estimates of different indicators and their respective 95% confidence intervals (95% CI) are presented. *P*-values <.05 values were considered statistically significant.

### 2.5 Role of the funding source

The funder had no role in manuscript development, data collection or analysis. The corresponding author had full access to the data presented and had final responsibility for the decision to submit for publication.

### 2.6 Ethical considerations

All the data analyzed were provided to PATH aggregated with no individual identifiers. No informed consent was requested as women were managed according to country based national guidelines.

## 3 Results

### 3.1 Screening

A total of 231,741 women were screened with HPV tests from May 2015 through December 2018. The target coverage of HPV-based screening, based on availability of HPV tests, was 91.6% in Guatemala, 88.7% in Nicaragua, 87.8% in Honduras, and 85.5% for the three countries combined in the selected project areas. Among all women reported to be screened with any screening option (Pap, VIA, or HPV) in the project target areas, HPV testing was the most common method used (59.7%), with 52.0% of screenings done with HPV testing in Guatemala (*N* = 85,226), 76.3% in Honduras (*N* = 72,873), and 52.3% in Nicaragua (*N* = 73,642). Nearly all women for whom age data were available fell within the country-specific target age range established for screening (97.7%) (Table 2). Among women for whom screening history was recorded, 55.8% of women in Nicaragua and 30.0% of women in Guatemala were screened for the first time. Information on screening history was not documented in Honduras. Overall, HPV was detected in 13.6% of women. HPV prevalence was significantly lower in Guatemala, 12.4% (95% CI: 12.2–12.6), as compared to 14.5% in Honduras (95% CI: 14.3–14.8) and 14.2% in Nicaragua (95% CI: 14.0–14.5) (Table 2).

**Table 2:** Characteristics of women who received HPV screening by country, in the scale-up project.

	Guatemala		Honduras		Nicaragua		Total all countries	
	N	%	N	%	N	%	N	%
Women screened for HPV	85,226	100	72,873	100	73,642	100	231,741	100
Method of sample collection Self-collected	56,693	90·2	20,349^b^	33·4	70,548	97·1	147,590	75·1
Clinician collected	6156	9·8	40,592	66·6	2139	2·9	48,887	24·9
*Data not collected*	*22,377*		*11,932*		*955*		*35,264*	
Included in target age group defined per country^a^								
Within target age range	60,542	96·6	68,193	97·8	72,542	98·5	201,277	97·7
Outside of target age range	2101	3·4	1505	2·2	1100	1·5	4706	2·3
*Data not collected*	*22,583*		*3175*		*0*		*25,758*	
Self-reported time since last cervical cancer screening								
Never screened	18,775	30·0	not reported	not reported	34,802	55·8	53,577	42·9
3 years or more	17,677	28·3	not reported	not reported	9383	15·0	27,060	21·7
<3 years	26,061	41·7	not reported	not reported	18,196	29·2	44,257	35·4
*Data not collected*	*22,713*		*72,873*		*11,261*		*106,847*	
Positive HPV screening result^c^ (95% CI)	10,557	12·4 (12·2–12·6)	10,589	14·5 (14·3–14·8)	10,476	14·2 (14·0–14·5)	31,622	13·6 (13·5–13·8)

CI: confidence interval.

a Target ages for screening with HPV by country: Guatemala: 30–65; Honduras: 30–64; Nicaragua: 30–59.

b Honduras offered self-sampling from 2017 to 2018 in the Tegucigalpa metro region only.

c All other test results were negative. Samples or plate runs that did not yield valid results were repeated until a result was obtained.

**Fig. 3:**
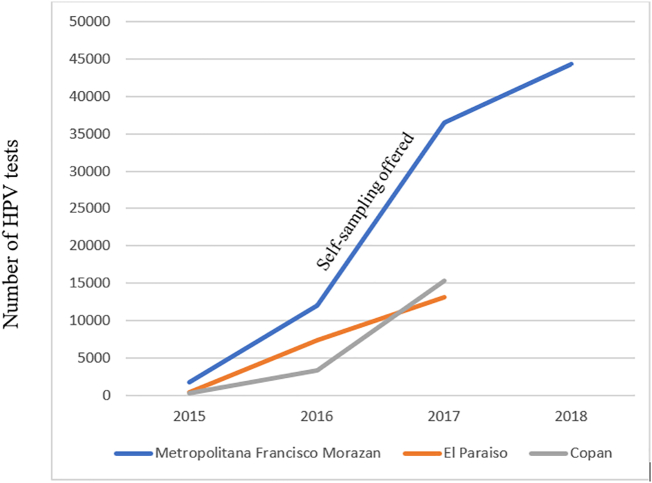
Cumulative number of HPV tests run in three regions in Honduras between 2015 and 18. Self-sampling was only implemented in Region Metropolitana de Francisco Morazan.

HPV screening through self-collected samples was high in Nicaragua (97.1%) and Guatemala (90.2%) and reached 74.0% in the metropolitan region of Tegucigalpa, Honduras, starting in 2017, considerably increasing the volume of tests performed in the area (Fig. 3).

### 3.2 Triage

As shown in Table 3, follow-up of HPV-positive women for triage and treatment varied significantly across countries. Overall, 73.7% of HPV-positive women completed a follow-up triage test to inform treatment decisions. The highest percentage of triage follow-up, 85.8%, occurred in Nicaragua (95% CI: 85.1–86.5) followed by Guatemala with 84.2% (95% CI: 83.4–84.9). Completion of triage in Honduras was substantially lower at 50.1% (95% CI: 49.2–51.1). More than one-third of women in Honduras, 38.5% (95% CI: 37.2–39.8), and 35.6% in Guatemala (95% CI: 34.6–36.6), were triage-positive primarily using VIA, whereas 27.5% (95% CI: 26.6–28.4) of women in Nicaragua were reported to have an abnormal Pap (Table 3), including atypical squamous cells of undetermined significance (ASC-US) and low-grade squamous intraepithelial lesion (LSIL+).

**Table 3:** Triage and treatment among HPV positive women within the public sector in the study areas.^a^

	Guatemala		Honduras		Nicaragua		Total all countries	
	N	% (95% CI)	N	% (95% CI)	N	% (95% CI)	N	% (95% CI)
Women HPV positive	10,557		10,833		10,476		31,866	
Women with follow-up:								
Women having a triage test (% among HPV positive)	8885	84·2 (83·4–84·9)	5432	50·1 (49·2–51·1)	8991	85·8 (85·1–86·5)	23,308	73·7 (72·7–73·6)
Women having a positive triage test (% among triaged)	3161	35·6 (34·6–36·6)	2091	38.5 (37·2–39·8)	2471	27·5 (26·6–28·4)	7723	33·1 (32·5–33·7)
Women treated^a^ (% among positive triage)	2676	84·7 (83·3–85·9)	1230	58·8 (56·7–60·9)	1657	67·1 (65·2–68·9)	5563	72·0 (71·0–73·0)

CI: confidence interval.

a Treatment refers to ablation procedures and does not always include women treated or managed in referral hospitals.

### 3.3 Treatment

Guatemala achieved a significantly higher proportion of HPV-positive, triage-positive women who completed treatment with 2676 (84.7%), followed by Nicaragua with 1657 (67.1%), and Honduras with 1230 (58.8%) (Table 3). Honduras had an important drop in treatment completion in 2017 but experienced an increase to 63% in 2018 when thermal ablation was introduced (data not shown).

Across the project countries, the reported *care*HPV test-failure rate was approximately 10%. Some plates had to be repeated due to suspected well-to-well contamination or due to unspecific test running failure (Camel et al., n.d.). The percent of failures remained consistent throughout the project, and therefore could not be solely attributable to a technical learning process.

## 4 Discussion

Within Scale-Up, nearly a quarter of a million women were screened for cervical cancer in a four-year project period across Guatemala, Honduras, and Nicaragua. By testing for oncogenic HPV types and using self-sampling the project addressed main barriers that have historically limited effectiveness of cervical cancer screening in low-resource settings. For the first time, these countries collected data on the proportion of screened women who received triage and treatment. Overall, two out of three screen-positive women received triage, and two out of three triage-positive women received treatment.

One of the major barriers to controlling cervical cancer in low-resource settings has been the difficulty of achieving high screening coverage. In an HPV-based screening approach, using self-collected samples is key to increasing coverage in low-resource settings. Self-sampling bypasses a pelvic exam and associated issues such as clinic access, provider availability and training, and patient discomfort (Silkensen et al., 2018; Cuzick et al., 2012). However, information on self-sampling advantages and accuracy of the approach need to be readily available to inform women and providers. Women may initially express concerns about their ability to collect an adequate sample or providers may not trust a vaginal sample for cervical cancer screening (Polman et al., 2019). In recent years, self-sampling is expanding in high-income settings like Canada, The Netherlands, USA, or Sweden, and also in LMICs like Argentina, Kenya, and Ghana, building the evidence base for the acceptability of this approach. Self-sampling approaches can reach a comparable sensitivity to clinician-collected samples when the test is polymerase chain reaction–based (Arbyn et al., 2018; Jeronimo et al., 2014). When a hybrid capture test such as *care*HPV is used, sensitivity is slightly reduced but is still higher than Pap or VIA.

The Scale-Up project demonstrated that use of HPV testing, including self-sampling, was acceptable and feasible to implement for a large volume of women across the three countries and achieved a high coverage between screened women and available tests. In Honduras, the annual number of women screened increased substantially in the third year of the project after the self-sampling option was extended to more women, and it remains part of the national guidelines when HPV tests are available (Figueroa, 2019). Further, the large proportion of women undergoing their first screening within the Scale-Up project suggests that self-sampling greatly improved outreach to women who had not previously participated in cervical cancer screening. If this level of effort could be sustained, a high population coverage in 5 years could be attained.

The HPV prevalence detected in the three countries was higher than that observed in screened populations in Europe or the US (Carozzi et al., 2012; Gage et al., 2015), but was consistent with expected values in the general population in the Latin American Region (Arrossi et al., 2019; Bruni et al., 2010), ranging from 12.4% to 14.5% in the target population of women 30 years and older. The differences in HPV prevalence across the Scale-Up countries were minimal in absolute terms and unlikely to represent a major variability in risk across these populations. The higher HPV prevalence in the Latin American region compared to North America or European populations (Bruni et al., 2010) of same age range may be related to a higher average number of sexual partners in the population but also to low screening coverage. Regular screening with high coverage is expected to identify and treat HPV related lesions leading to long term progressive reduction of the background HPV prevalence in adult women (Landy et al., 2020) A large part of the study population included in the Scale-up project were newly screened. Although high-risk groups, like sex workers, were included in the Scale-up screening process, these were, to our knowledge, small in size, and thus our data reflect largely the HPV prevalence of the general population.

PATH selected the *care*HPV test, now prequalified by WHO (World Health Organization (WHO) website, n.d.-b), for the Scale-Up project in 2014 because its low cost and moderate technical complexity were attractive characteristics for long-term sustainability beyond the duration of the project period. Experiences with *care*HPV have been inconsistent across studies, with some showing good performance (Segondy et al., 2016; Poli et al., 2018)and management (Poli et al., 2018) and others reporting implementation issues, such as a steep learning curve for technicians and high percentage of invalid plates (Umulisa et al., 2018). Testing with *care*HPV requires a certain level of training of technical personnel. It is not a point-of-care test, and samples must be run in batches of 90 to minimize waste. Approximately 10% of tests executed for the current project did not generate a result. Reasons were diverse, but included lab technician error, power outages, malfunction of the test system, and suspected well-to-well contamination (Beylerian et al., 2018). A detailed analysis of these issues is ongoing in Guatemala and Honduras (manuscript in preparation).

It has been recently reported that in Papua New Guinea, the use of an HPV test for primary screening resulted in higher accuracy in treating high-grade lesions (92%) with an overtreatment rate of 13% (Toliman et al., 2018). In that setting, adding VIA as a triage test halved the number of women sent for treatment and reduced overtreatment to 3.7%, but the test accuracy dropped to 45.5%. These trade-offs need to be carefully considered when updating or introducing new screening algorithms.

Any gap in time between diagnosis and treatment creates opportunity for loss to follow-up whereas women with a positive HPV test through self-sampling had to be recalled to a clinic for triage. In El Salvador, women under a screen-and-treat protocol had approximately twice the treatment completion rate compared to women referred to colposcopy (Cremer et al., 2017). In Scale-Up, while major efforts were devoted to screen women; follow-up and treatment were less systematic. The one-visit approach, where women are screened, triaged, and treated on the same day is a preferable option but is challenging given need for capacity within screening sites to triage and treat women. Recent data suggest that automated visual evaluation (AVE) based on artificial intelligence–generated algorithms can be used on digital images collected during the gynecological examination, improving the prediction of CIN3+ lesions with higher accuracy than HPV, VIA, or Pap tests (Hu et al., 2019). Active research aiming to reach a low-cost and highly-accurate approach for screening or triage of HPV-positive women is ongoing in this area (*Controlling Cervical Cancer: DCEG's Ongoing Commitment to Improving Women's Health [Internet*], 2019).

Similarly, the introduction of thermal ablation for same-day treatment contributes substantially to higher treatment percentages (Basu et al., 2018). We commonly observed challenges to consistent access to cryotherapy in the Scale-Up countries. Thermal ablation is now expanding in many settings; it is prequalified by WHO, devices can be easily transported between clinics, and it is available at an accessible cost (Randall et al., 2019; *WHO Guidelines for the Use of Thermal Ablation for Cervical Pre-cancer Lesions*, 2019). During our project, thermal ablation was introduced in Guatemala and Honduras with an overall high acceptability (Sandoval et al., 2019).

Any cervical-cancer screening program should aim to reduce cervical cancer mortality and thus requires monitoring measures of effectiveness. An estimated 85% of treatment-eligible women must be treated for precancerous lesions to show an impact at the population level (Campos et al., 2017). Ethically, every woman detected through a screening process should be managed adequately and treated according to evidence-based guidelines. Few data were available before the Scale-Up project on screening coverage and treatment. Murillo et al. (Murillo et al., 2008) reported that the screening coverage under the national guidelines was 26.4% in Guatemala, 41.2% in Honduras and 23.3% in Nicaragua. None of these countries reported changes in mortality of cervical cancer, suggesting poor impact of screening activities (Murillo et al., 2008). However, MOH personnel accounts, together with incidence rates of cervical cancer, indicate that these countries had long suffered low treatment access prior to the project. Among the estimated residents in the Scale-Up target areas, around 18% of the women in Guatemala and Honduras, and 59% in Nicaragua, were screened during the project period and Scale-Up partners have continued their efforts to recall women who were lost to follow up. These actions are expected to have an impact in mortality in the years to come.

There are myriad challenges to delivering HPV screening results to women and engaging HPV-positive women to complete triage and treatment if needed; these include limited longitudinal health information systems, requiring women to appear for multiple appointments and travel to specialized facilities, lack of trained providers able to provide follow-up, and lack of treatment availability. Under the Scale-Up project, providers from primary health care centers involved in the project recommended that women return to the clinic for results approximately one month after screening, but there was no call-and-recall system established to reinforce follow up which can easily derive in losing effectiveness of the screening activities.

Although mobile phones are widely available, no specific communication was undertaken at the primary health care level to recall women. Many settings are now exploring how best use the mobile technology through automatic or directed SMS as a way for women to keep in regular contact with medical services (Linde et al., 2017; Moodley et al., 2019). Inclusion of treatment completion as a monitoring indicator is key to assess adequate follow-up strategies, as measuring screening uptake alone not comprehensive enough. If the health information system could identify, in an automatic way, women that are screen- positive and that need any type of follow-up or treatment, health centers could react more easily to these lists. Otherwise, they remain disconnected with the screening outcomes unless women request the follow up.

The Scale-Up project was not designed to report on coverage or population-level impact, but we could estimate the capacity of the system by reporting the target coverage performance which was high. Individual-level data were not available to the Scale-Up project and duplicate testing or shorter intervals between screening tests were not measured; a limited set of individual data from the MOH of Honduras and Nicaragua were retrieved for additional quality control checks. Further, access to pathology records was not available for Scale-Up and no population-based cancer registry exist to measure of impact. Honduras is now in the early stages of developing a population-based registry that will reduce this limitation in the near future.

## 5 Conclusion

The Scale-Up project successfully launched and implemented a new cervical cancer screening strategy in a high-burden region. The project worked with three Central American MOHs to update national guidelines, train local staff, and engage local champions to introduce key innovations in screening (molecular testing, self-sampling, use of a focused set of key indicators) and improve knowledge of and capacity for clinical and laboratory management of HPV tests. Efforts to gather specific indicators were important for project evaluation and accountability to stakeholders. At the time of completing this manuscript, the MOHs of Guatemala and Honduras are planning to continue with HPV testing, while the MOH of Nicaragua intends to re-initiate and expand HPV testing at a future date. The limited quality of available triage approaches remains a challenge, but new approaches with the potential to improve visual evaluation of the cervix for both screening and triage purposes will likely be available soon (Hu et al., 2019).

The Scale-Up project provides evidence that moving toward the elimination of cervical cancer with high-precision screening is possible in low-resource settings. Yet this will only be attainable if, in addition to primary prevention through HPV vaccines, well-organized efforts for follow up are used at each screening step. Scale-Up countries have generated encouraging evidence that HPV testing, self-sampling, and thermal ablation can overcome many previous obstacles to screening and timely treatment, resulting in high volume of women screened and treated for cervical precancer and cancer lesions in LMICs.

## CRediT authorship contribution statement

**Francesca Holme:**Conceptualization, Visualization, Formal analysis, Data curation, Writing - original draft, Writing - review & editing.**Jose Jeronimo:**Conceptualization, Data curation, Writing - review & editing.**Francisco Maldonado:**Conceptualization, Visualization, Data curation, Writing - review & editing.**Claudia Camel:**Conceptualization, Visualization, Data curation, Writing - review & editing.**Manuel Sandoval:**Conceptualization, Visualization, Data curation, Writing - review & editing.**Benito Martinez-Granera:**Conceptualization, Data curation, Writing - review & editing.**Mirna Montenegro:**Conceptualization, Data curation, Writing - review & editing.**Jacqueline Figueroa:**Conceptualization, Data curation, Writing - review & editing.**Rose Slavkovsky:**Visualization, Data curation, Writing - review & editing.**Kerry A. Thomson:**Writing - original draft, Writing - review & editing.**Silvia de Sanjose:**Visualization, Formal analysis, Data curation, Writing - original draft, Writing - review & editing.

## Declaration of competing interest

Francesca Holme, Francisco Maldonado; Claudia Camel; Manuel Sandoval; Benito Martinez-Granera, Mirna Montenegro, Jacqueline Figueroa, Rose Slavkovsky, Kerry A. Thomson and Silvia de Sanjose declare no competing interests.

Jose Jeronimo was the co-owner and Deputy Manager of Onco Prev International, a Peruvian company, from 2012 through March 2017. Onco Prev offers cervical cancer screening services and in 2016 also began positioning for distribution of medical devices including colposcopes and the Liger thermo-coagulator. Onco Prev International did not commercialize any medical instrument during the time JJ was part of that company. JJ declares that he has never received an honorarium, travel money, or compensation from any company manufacturing thermal ablation equipment.
